# Voluntary Physical Activity Modulates Brain ΔFOSB and Alters Coactivation Networks in Male and Female Mice

**DOI:** 10.1523/ENEURO.0066-26.2026

**Published:** 2026-10-01

**Authors:** Marene H. Hardonk, Rick Wenning, Jazz Stofberg, Meike H. Mulder, Anna H. Vuuregge, Jorine Geertsema, Susanne E. la Fleur, Paul J. Lucassen, Joram D. Mul

**Affiliations:** ^1^Brain Plasticity group, Swammerdam Institute for Life Sciences, Faculty of Science, University of Amsterdam, Amsterdam 1098 XH, The Netherlands; ^2^Centre for Urban Mental Health, University of Amsterdam, Amsterdam 1012 GC, The Netherlands; ^3^Laboratory of Endocrinology, Department of Laboratory Medicine, Amsterdam UMC, University of Amsterdam, Amsterdam 1105 AZ, The Netherlands; ^4^Amsterdam Gastroenterology, Endocrinology and Metabolism (AGEM), Amsterdam 1105 AZ, The Netherlands; ^5^Netherlands Institute for Neuroscience, Institute of the Royal Netherlands Academy of Arts and Sciences (KNAW), Amsterdam 1105 BA, The Netherlands; ^6^Amsterdam Neuroscience, Mood Anxiety Psychosis Stress and Sleep, Amsterdam 1081 HV, The Netherlands

**Keywords:** anxiety, brain health, cognition, depression, stress resilience, voluntary wheel running

## Abstract

Regular physical activity promotes brain health, yet the underlying mechanisms remain incompletely understood. Repetitive activation of neurons results in accumulation of the transcription factor ΔFOSB, a long-lived splice variant of FOSB. Long-term voluntary wheel running (VWR), a behavioral paradigm that mimics human exercise training, altered brain ΔFOSB immunoreactivity signatures and reorganized coactivation networks in Wistar rats. Here, we used a similar approach to determine large-scale ΔFOSB brain signatures following long-term VWR in mice. Young-adult individually housed male and female C57BL/6J^OlaHsd^ mice were allowed to run for 4 weeks on horizontal saucer-like wheels, after which ΔFOSB immunoreactivity was quantified in 46 brain regions associated with stress regulation and cognition- and reward-related behavior. Network analysis was applied to assess VWR-mediated changes in interregional ΔFOSB coactivation patterns and network topology. Male and female mice ran equal distances, and VWR blunted body weight gain and terminal gonadal white adipose tissue mass in both sexes. VWR modulated ΔFOSB immunoreactivity across several cortical, striatal, hippocampal, and thalamic regions. Network analysis revealed substantial network reorganizations, with reduced overall network density and increased cortical centrality in males, and greater global efficiency (i.e., small-worldness) in females. Thus, VWR induced large-scale adaptations in brain (in)activation, reshaping network organization in distinct ways in both sexes. Because ΔFOSB regulates many target genes, impacting, e.g., neuron excitability, our findings suggest that long-term VWR induces widespread transcriptional alterations throughout the mouse brain. Functional and mechanistic follow-up studies are necessary to determine the impact of these alterations on stress regulation and cognition- and reward-related behavior.

## Significance Statement

Regular physical activity promotes brain health, but the underlying mechanisms remain incompletely understood. Here, we quantified ΔFOSB, a transcription factor involved in neuroplasticity, in 46 brain regions associated with stress regulation and cognition- and reward-related behavior after 4 weeks of voluntary wheel running (VWR) in individually housed male and female mice. We show altered ΔFOSB immunoreactivity in a subset of these brain regions in both male and female runners. This was accompanied by specific changes in ΔFOSB coactivation networks. These large-scale mouse brain ΔFOSB signatures following VWR improve our understanding of how VWR impacts brain plasticity in mice and offer a framework for mechanistic and functional studies into ΔFOSB-mediated changes in stress regulation and cognition- and reward-related behavior following VWR.

## Introduction

Regular physical activity provides many benefits for brain health, including enhanced neuroplasticity, a lower risk for neurodegenerative and mental health disorders, and positive effects on cognition and substance use. However, the brain mechanisms underlying these benefits are still not well understood. Clarifying the neurobiological processes that link exercise training to improvements in stress regulation, cognition, and reward-related behavior in males and females may help refine future physical activity-based treatments to support brain health, including mental health, cognitive function, reward-related behavior, and healthy aging.

Mechanistic studies in rodents have identified the transcription factor ΔFOSB as a key molecular driver of neuroplasticity and long-term adaptations across a wide range of conditions, including depression, antidepressant treatment, drug addiction, Parkinson's disease therapy, Alzheimer's disease, adult hippocampal neurogenesis, epilepsy, and learning and memory (Mandelzys et al., 1997; Berton et al., 2009; Vialou et al., 2010b; Robison and Nestler, 2011, 2022; Yutsudo et al., 2013; Eagle et al., 2015, 2016, 2018, 2020; Nestler, 2015; Corbett et al., 2017; Manning et al., 2017, 2019; You et al., 2017; Beck et al., 2019; Lamothe-Molina et al., 2022). ΔFOSB, part of the FOS family of immediate early genes, is a truncated splice variant of FOSB that can form self-assemblies or activator protein-1 (AP-1) transcriptional complexes with JUN proteins to regulate temporally dependent transcription of a broad set of target genes (Hope, 1998; McClung and Nestler, 2003; Nestler, 2015; Yin et al., 2020). This transcriptional activity impacts neuron function, as viral-mediated overexpression of ΔFOSB in the mouse dorsal (dHPC) or ventral hippocampus (vHPC) decreased neuronal excitability (Eagle et al., 2018, 2020) and high levels of ΔFOSB are associated with repression of cFOS, a marker of acute neuronal activation (Lamothe-Molina et al., 2022). Finally, ΔFOSB has a well-established long half-life (Chen et al., 1997; Carle et al., 2007; Ulery-Reynolds et al., 2009), allowing it to accumulate in neurons, in particular following repetitive activation (Hope, 1998; Kelz and Nestler, 2000; Nestler et al., 2001; Andersson et al., 2003; Kreuter et al., 2004; Cates et al., 2014). This makes ΔFOSB particularly suitable to quantify patterns of chronic neuronal activation, for example, during long-term voluntary wheel running (VWR).

We recently used this ΔFOSB quantification approach in male and female Wistar rats and reported large-scale VWR-mediated changes in ΔFOSB immunoreactivity in various brain regions involved in stress regulation, cognition, and reward-related behaviors compared with sedentary control rats, as well as changes in ΔFOSB coactivation networks (Hardonk et al., 2026). Here, we extend this large-scale approach to mice and quantified ΔFOSB immunoreactivity in 46 brain regions associated with stress regulation and cognition- and reward-related behavior in male and female C57BL/6J^OlaHsd^ mice following 4 weeks of VWR. Subsequently, we constructed ΔFOSB coactivation networks to assess how VWR reorganizes brain neuronal activity network patterns.

## Methods and Materials

### Ethical approval and monitoring

All experimental procedures were performed in accordance with the European guidelines for laboratory animals (EU directive 2010/63/EU) and approved by the Dutch Central Committee for Animal Experiments (CCD; AVD8010020172424) and the Agency for Animal Welfare (IvD) of the Netherlands Institute of Neuroscience (NIN; Royal Dutch Academy of Sciences), Amsterdam, and the IvD of the University of Amsterdam, Amsterdam, as required by Dutch law.

### Animals and housing conditions

A graphical overview of the experimental procedures is presented in Figure 1. For this study, we used 24 male (19–22 g and ∼6 weeks of age at arrival) and 24 female (18–22 g and ∼12 weeks of age at arrival) C57BL/6J^OlaHsd^ mice (strain code, 057, Inotiv). Average litter size for the C57BL/6J^OlaHsd^ inbred strain at this commercial breeder is 5.0 pups/litter (Inotiv). Males and females were studied in two separate cohorts (*n* = 24/cohort). Mice were housed at the animal facility of the Swammerdam Institute for Life Sciences at the University of Amsterdam in a temperature- (21–23°C), humidity- (40–60%), and light-controlled room (12:12 h light/dark cycle) with lights on at zeitgeber time 0 (ZT0; 08:00).

Mice were allowed to recover from transport stress and left undisturbed for 7 d during group housing (four animals/cage) in high-temperature polysulfane (PSU) type II long cages measuring 36.5 × 20.7 × 14.0 cm (length × width × height; 530 cm^2^, 1284L Eurostandard Type II L, Tecniplast) with standard wood-chip bedding and *ad libitum* access to a bottle of tap water and a pelleted diet [#801722, CRM (P), 22% protein, 69% carbohydrate, and 9% fat by energy, 3.6 kcal/g gross energy, Special Diet Services], unless mentioned otherwise, and with a wooden gnawing stick, and Enviro-Dri nesting material as cage enrichment. Continuous and soft radio sound provided background noise.

### Energy homeostasis and VWR

Following acclimatization to the animal facility, RandoMice (Van Eenige et al., 2020) was used to randomly assign mice based on body weight to either one of the two experimental groups: runners (RUN; male, *n* = 12; female, *n* = 12) and sedentary controls (SED; male, *n* = 12; female *n* = 12). Sample sizes were calculated to ensure power (>0.8) and were based on prior experience with this behavioral paradigm as well as previous experiments of comparable design. All mice were housed individually in PSU type II long individually ventilated cages (IVC; 39.6 × 21.5 × 17.2 cm, 542 cm^2^, 1285L cage, Tecniplast) with the previously mentioned cage enrichment (a wooden gnawing stick and Enviro-Dri nesting material) as well as a red mouse hut (Bio Services) and a locked low-profile wireless inclined (horizontal) running wheel (15.5 cm diameter, 10.16 cm high, Med Associates, ENV-047 and ENV-044 for RUN and SED groups, respectively) and *ad libitum* access to a bottle of tap water and the CRM (P) diet for a baseline period of 7 d.

Following this baseline period, wheels were unlocked for only the RUN group for another 28 d. Wheel revolutions per cage were recorded per minute using the accompanying software provided by the manufacturer (Wheel Manager Data Acquisition Software, SOF-860, Med Associates). Total distance run was calculated based on the total number of revolutions and the circumference traveled for each revolution [12.04 cm/revolution, estimated from the external (15.5 cm) and internal (9 cm) diameter of the running wheels]. Throughout baseline and the VWR period, body weight, food intake, and water intake were measured weekly. After 28 d of running, the wheels of the RUN group were locked at the start of the light phase for 24 h before harvesting brain material. This was done to allow the degradation of any residual full-length (regular) FOSB protein, such that all remaining immunoreactivity reflects the accumulated ΔFOSB signal (Nestler et al., 2001; Perrotti et al., 2004).

Blinding during the experiment was not feasible due to the presence of an unlocked or locked running wheel; however, all data analyses were conducted by investigators who were blinded to the group identity. Two male runners were excluded from the experiment due to health-related complications of unknown origin: one animal was found dead in the home cage, and the other animal exhibited impaired health and a marked cessation of running behavior after 1 week and was therefore removed from the experiment. Data from these two mice were excluded from all analyses. One female runner was excluded from running wheel analyses, and several regions of interest (ROIs) of two sedentary animals were excluded from all ΔFOSB-related analyses due to technological failures (i.e., failed running wheel recordings and unsuccessful ΔFOSB staining, respectively).

### Tissue collection and single-labeling immunohistochemistry

After a 24 h wheel blockade, mice were fasted starting at ZT0 and killed between ZT0 and ZT8 via an overdose of intraperitoneally administered pentobarbital (Euthasol; 120 mg/kg). Gonadal white adipose tissue (gWAT) pads and adrenals were bilaterally removed and weighed prior to transcardial perfusion with ice-cold 0.9% saline (0.9% w/v NaCl in ultrapure H_2_O) followed by ice-cold 4% paraformaldehyde (PFA) in 0.1 M phosphate buffer (PB), pH 7.4. Afterward, brains were dissected and postfixed in 4% PFA in PBS for 24 h at 4°C and then washed in PBS and transferred to a 0.01% w/v sodium azide in 0.1 M PB solution and stored at 4°C until use.

Prior to sectioning, brains were cryoprotected in 15% (w/v) sucrose in 0.1 M PB at 4°C until saturated (i.e., completely sunken to the bottom of the Falcon tube), followed by overnight immersion in 30% (w/v) sucrose in 0.1 M PB at 4°C until saturation. Coronal brain sections (35 μm) were cut using a sliding microtome (Jung HN40, Leica Biosystems) and stored in cryoprotectant solution (20% v/v glycerol, 30% v/v ethylene glycol in 0.05 M PBS) at −20°C. Sections containing the cortex, basal ganglia/septum, hippocampus, (extended) amygdala, thalamus, hypothalamus, and midbrain were collected from bregma +2.80 mm to bregma −4.04 mm (Paxinos and Franklin, 2001).

Visualization of ΔFOSB signal was performed as previously described (Hardonk et al., 2026). In short, free-floating sections from male brains were washed in 0.05 M Tris-buffered saline (TBS), endogenous peroxidase activity was inhibited by pretreating for 10 min with 1.5% v/v H_2_0_2_ (Merck; in TBS) before incubation with rabbit anti-FOSB [5G4; #2251, Cell Signaling Technology, RRID: AB_2106903; 1:1,000; this antibody recognizes FOSB, ΔFOSB, variant FOSB (vFOSB), and Δ2ΔFOSB in striatal nuclear extracts, which are all absent in *fosB*-null mice (Ohnishi et al., 2011)] in supermix (0.25% w/v gelatin, 0.5% v/v Triton X-100, in 1× TBS), pH 7.6, for 1 h at room temperature (RT) and overnight at 4°C. Following incubation with a biotinylated goat anti-rabbit IgG (H + L; BA-1,000; Vector Laboratories, RRID: AB_2313606; 1:200 in supermix) for 2 h at RT, signal amplification was achieved using avidin–biotin complex (Vectastain Elite ABC HRP Kit, PK-6100, Vector Laboratories; 1:800 in supermix) for 1.5 h at RT and visualized by nickel-enhanced DAB [0.05% w/v 3.3-diaminobenzidine (Sigma-Aldrich), 0.23% w/v nickel-ammonium sulfate (Merck), and 0.01% w/v H_2_O_2_ (Merck) in 0.05 M TB], pH 7.6. Sections were then mounted onto SuperFrost Plus slides (Thermo Fisher Scientific), air-dried, dehydrated through graded ethanol, and cleared in xylene, before slides were coverslipped with Entellan (#107961, Merck). For female brain sections, initial staining using the same protocol as for male brain sections resulted in suboptimal ΔFOSB immunoreactivity, limiting quantitative analysis. Antigen retrieval was therefore applied to sections from this female cohort only in order to facilitate quantifiable ΔFOSB staining similar to the levels observed in males. Sections were then mounted on SuperFrost Plus slides, outlined with a PAP-PEN liquid blocker and dried overnight. Slides were washed in TBS, and antigen retrieval was performed by microwaving the slides in 0.05 M TB, pH 9.0, for 15 min (800 W, heat reduced to 240 W when boiling). After cooling for 30 min, ΔFOSB immunohistochemistry was performed as described above. Male and female brains were otherwise processed identically.

### Image acquisition and analysis

For quantification of ΔFOSB-positive nuclei and construction of coactivation networks, an experimental pipeline was used similar to that previously described (Hardonk et al., 2026). In short, randomly numbered coronal brain sections were scanned at 10× bright-field magnification using a Zeiss Axio Scan Z1 slide scanner (Carl Zeiss) to ensure blindness to group identity during analysis. ΔFOSB-expressing cells per ROI were quantified using a modified version of the atlas-based analysis (Bourgeois et al., 2021) in FIJI (ImageJ2), which was adapted in-house for bilateral mouse brain sections. Sections were converted to 8 bit grayscale, aligned to a mouse brain atlas (Bourgeois et al., 2021), and thresholded [Robust Automatic Threshold Selection plugin (noise = 25, lambda = 3); Analyze Particle function (size = 20–250, circularity = 0.40–1.00)] to detect strongly labeled ΔFOSB-positive nuclei. Counts were normalized to ROI area and mean of the respective sedentary control group. Missing or damaged ROIs were excluded. An overview of included ROIs and full regional details are provided in Figure 1*C* and Extended Data Figures 3-1 and 4-1.

### Coactivation network generation and permutation analyses

Coactivation network analyses were conducted as previously described (Terstege and Epp, 2022; Hardonk et al., 2026). Pearson’s correlations between regional ΔFOSB immunoreactivity levels were calculated in R (version 4.4.1) to generate group-specific correlation matrices and were visualized as unweighted (i.e., unthresholded) and weighted (i.e., thresholded) heatmaps. From these matrices, coactivation networks were constructed by thresholding correlations at *p* < 0.005 using the igraph package (version 2.1.4), with brain regions represented as nodes and significant correlations as edges.

To further describe the coactivation networks, global (i.e., density, small-worldness) and local (i.e., degree, weighted betweenness centrality) network metrics were computed using the igraph package following established methods (Watts and Strogatz, 1998; Newman, 2003; Onnela et al., 2005; Rubinov and Sporns, 2010; Bullmore and Bassett, 2011). To assess whether VWR or sex reorganized the overall network architecture, similarity between the two correlation matrices of interest was evaluated using a Mantel test with 999 permutations. To identify which specific interregional correlations were altered, correlations from one group were subtracted from the other, and group differences in correlation coefficients were evaluated using permutation testing (*n* = 1,000; Jin et al., 2025).

### Statistical analysis

All behavioral, metabolic, and physiological data were analyzed using two-tailed unpaired Student's *t* tests or Mann–Whitney *U* tests when parametric assumptions were violated and two-way ANOVAs with repeated measures where appropriate, followed by Šidák post hoc tests. Regional ΔFOSB analyses included false discovery rate (FDR) correction for multiple comparisons using the Benjamini–Hochberg procedure, with an FDR threshold of *P*_corrected_ < 0.05, and both uncorrected and FDR-corrected *p* values are reported. Correlations were assessed using simple linear regression. For all behavioral, metabolic, and physiological data, as well as regional ΔFOSB analyses, statistical analyses and data visualization were conducted in GraphPad Prism (v10.2.3), and *p* < 0.05 was considered significant, while *p* < 0.10 was considered a trend toward an effect. See all figure legends and Extended Data Figures 3-1 and 4-1 for full statistical details.

Interregional ΔFOSB immunoreactivity correlations were computed and visualized in unfiltered heatmaps. Networks were constructed and analyzed based on thresholded correlation matrices (*p* < 0.005). Overall matrix differences between groups were assessed using a Mantel test with 999 permutations, with *p* < 0.05 considered statistically significant. Permutation testing of individual correlation differences between experimental groups was performed using bootstrapping (*n* = 1,000), with unweighted and significant results (*p* < 0.005) visualized in heatmaps and volcano plots (with an additional threshold for correlation differences of >1). All network analyses, statistical computations, and visualizations were performed in R (version 4.4.1).

## Results

### Long-term VWR behavior

To examine how long-term VWR influenced ΔFOSB immunocytochemical staining levels and brain coactivation networks, young-adult male and female mice were housed individually with either a locked running wheel (controls) or with an unlocked wheel for 28 d (runners; Fig. 1*A*). Both sexes gradually increased their daily running distances over the first days, after which running activity stabilized at ∼13 km/d in males and 17 km/d in females (Fig. 2*A*,*B*). Across the 28 d period, males and females ran a total distance of 351.2 ± 38.3 km and 432.5 ± 24.9 km on average, respectively, with no significant differences between sexes (Fig. 2*C*). As expected with a nocturnal species, most running occurred during the dark phase (Extended Data Fig. 2-1*A*–*J*). Overall, both males and females displayed robust habitual VWR.

**Figure 1.**
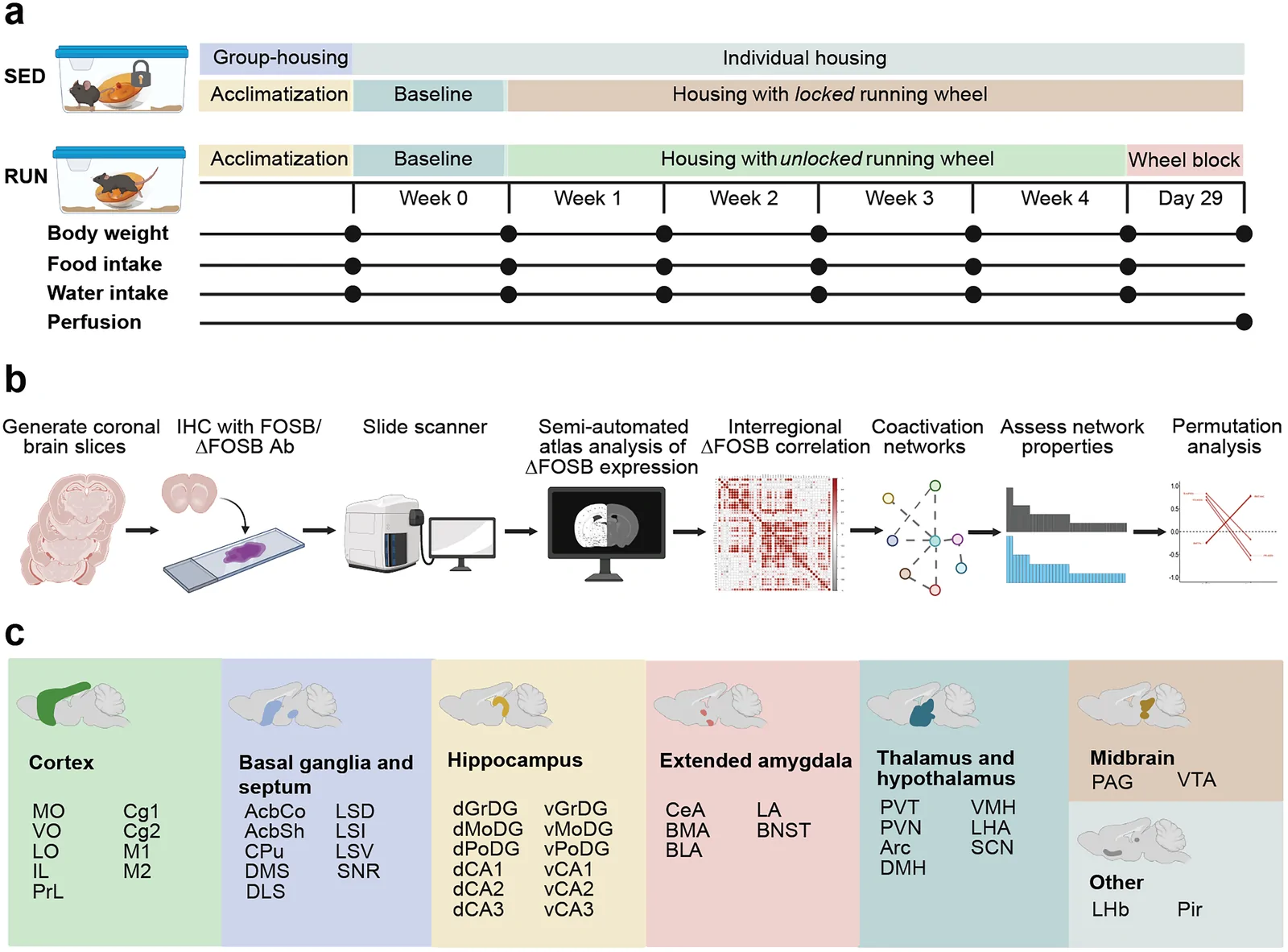
Experimental timeline and workflow. ***a***, Experimental timeline of animal studies. All male and female mice were housed with a locked running wheel (sedentary; SED) during the 7 d baseline period. During the subsequent 28 d experimental period, SED controls remained housed with a locked running wheel, whereas the running wheels of runners (RUN) were unlocked. Body weight and food and water intake were measured weekly. Wheels were blocked for 24 h before isolation of brains. ***b***, Experimental workflow of immunohistochemistry, ΔFOSB quantification, and ΔFOSB coactivation network generation and analysis. ***c***, Brain ROIs categorized by parent structures: cortex (green), basal ganglia and septum (purple), hippocampus (yellow), extended amygdala (pink), thalamus and hypothalamus (blue), midbrain (orange), and other (gray). Adapted from Hardonk et al. (2026).

**Figure 2.**
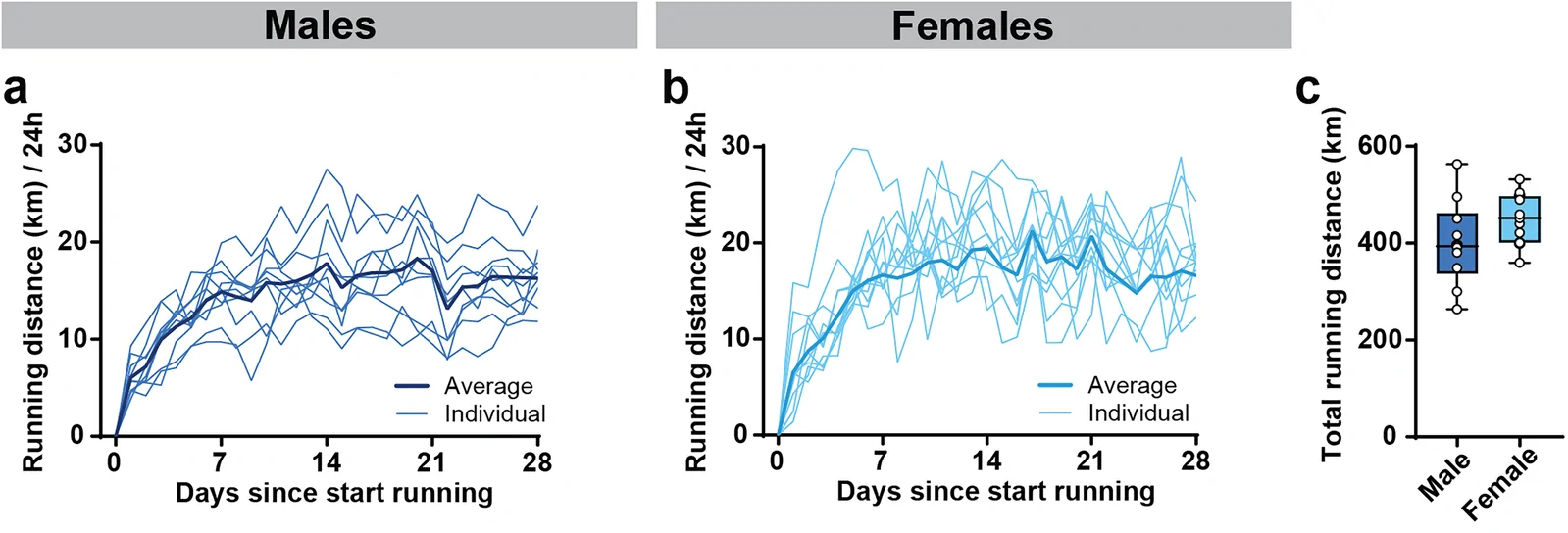
Running behavior in individually housed male and female C57BL/6J^OlaHsd^ mice. ***a*, *b***, Daily individual and average running distances of individually housed (***a***) male and (***b***) female C57BL/6J^OlaHsd^ mice. ***c***, Total running distance of males and females during 28 d of running (*t*_(19)_ = 1.593, *p* = 0.128). Data are presented as the mean with lines representing individual mice (***a***, ***b***) or as box plots indicating the median (line) and mean (+), the interquartile range, and the minimum to maximum values of the data distribution, with dots representing individual mice (***c***). (***a*–*c***) *n* = 10–11/group. Circadian dynamics of daily VWR are shown in Extended Data Figure 2-1. The impact of long-term VWR on body weight and food and water intake is shown in Extended Data Figure 2-2. Impact of long-term VWR on several physiological markers is shown in Extended Data Figure 2-3.

10.1523/ENEURO.0066-26.2026.f2-1 Figure 2-1 **Circadian dynamics of daily VWR in individually-housed male and female C57BL/6J^OlaHsd^ mice.** (**a**-**h**) 24 h running patterns averaged per running week or (**i**, **j**) averaged for all running weeks (**a**, **c**, **e**, **g**, **i**) in males and (**b**, **d**, **f**, **h**, **j**) females. Zeitgeber Time (ZT) refers to time of day relative to the time of lights off/lights on, with lights off at ZT12. Data are presented as mean ± S.E.M. (a-j). (a-j) *n* = 10-11/group. Download Figure 2-1, TIF file.

10.1523/ENEURO.0066-26.2026.f2-2 Figure 2-2 **Impact of long-term VWR on body weight, food and water intake in individually-housed male and female C57BL/6J^OlaHsd^ mice.** (**a**) Body weight (BW) evolution (*time x housing* interaction, F_(5, 100)_ = 14.15, *P* < 0.0001; Šidák post hoc: *P < 0.05, SED *versus* RUN) and (**b**) total BW gain (t_(20)_ = 5.435, ****P < 0.0001 SED *versus* RUN) in male sedentary (SED) controls and runners (RUN). (**c**) BW evolution (*time x housing* interaction, F_(5, 110)_ = 2.074, *P* = 0.074) and (**d**) total BW gain (t_(22)_ = 0.469, *P* = 0.644 SED *versus* RUN) in female SED and RUN mice. (**e**) Average daily control diet (CD) intake (*time x housing* interaction, F_(4, 80)_ = 11.78, *P* < 0.0001; Šidák post hoc: ****P < 0.0001, ***P < 0.001, *P < 0.05, SED *versus* RUN) and (**f**) total CD intake (t_(20)_ = 1.180, *P* = 0.252 SED *versus* RUN) in male SED and RUN mice. (**g**) Control diet (CD) intake evolution (*time x housing* interaction, F_(4, 88)_ = 14.59, ****P < 0.0001; Šidák post hoc: **P < 0.01, SED *versus* RUN) and (**h**) total CD intake (t_(22)_ = 2.635, *P* = 0.015 SED *versus* RUN) in female SED and RUN mice. (**i**) Average daily water intake (*time x housing* interaction, F_(4, 79)_ = 8.390, ****P < 0.0001) and (**j**) total water intake (t_(20)_ = 0.932, *P* = 0.363 SED *versus* RUN) in male SED and RUN mice. (**k**) Average daily water intake (*time x housing* interaction, F_(4, 88)_ = 8.248, ****P < 0.0001) and (**l**) total water intake (t_(22)_ = 0.122, *P* = 0.904 SED *versus* RUN) of female SED and RUN mice. Data are presented as the mean ± S.E.M. (a, c, e, g, i, k) or as box plots indicating the median (line) and mean (+), the interquartile range, and the minimum to maximum values of the data distribution, with dots representing individual mice (SED = gray, RUN = blue; b, d, f, h, j, l). (a-l) *n* = 12/group. Download Figure 2-2, TIF file.

10.1523/ENEURO.0066-26.2026.f2-3 Figure 2-3 **Physiological impact of VWR in individually-housed male and female C57BL/6J^OlaHsd^ mice. (a)** Terminal gonadal white adipose tissue (gWAT) weight, normalized for body weight, in male sedentary (SED) controls and runners (RUN; t_(20)_ = 7.652, *P* < 0.0001 SED *versus* RUN) and (**b**) correlation between terminal gWAT weight and total running distance (R² = 0.531, F_(1,8)_ = 9.047, *P* = 0.017) in male RUN mice. (**c**) Terminal gWAT weight, normalized for body weight, in female SED and RUN mice (t_(22)_ = 3.384, *P* = 0.003 SED *versus* RUN) and (**d**) correlation between terminal gWAT weight and total running distance (R² = 0.296, F_(1,9)_ = 0.396, *P* = 0.600) in female RUN mice. (**e**) Terminal adrenal weight (t_(20)_ = 1.222, *P* = 0.236 SED *versus* RUN) in male SED and RUN mice and (**f**) correlation between terminal adrenal weight and total running distance (R² = 0.084, F_(1,8)_ = 0.737, *P* = 0.416) in male RUN mice. (**g**) Terminal adrenals weight (t_(22)_ = 0.070, *P* = 0.945 SED *versus* RUN) in female SED and RUN mice and (**h**) correlation between terminal adrenal weight and total running distance (R² = 0.234, F_(1,9)_ = 2.742, *P* = 0.132) in female RUN mice. Data are presented as box plots indicating the median (line) and mean (+), the interquartile range, and the minimum to maximum values of the data distribution, with dots representing individual mice (a, c, e, g) or as regression plots indicating the simple linear regression (line) with dots representing the individual mice (b, d, f, h). (a-h) *n* = 10-12/group. Download Figure 2-3, TIF file.

### Physiological and metabolic impact of long-term VWR

Both male and female mice, irrespective of running condition, gained body weight during the experimental period. However, male runners exhibited significantly lower body weight gain compared with sedentary controls from Week 2 onward, whereas no significant differences were detected between female runners and sedentary controls during the entire VWR period (Extended Data Fig. 2-2*A*–*D*). Thus, VWR attenuated body weight gain exclusively in males.

Running reduced daily caloric intake compared with sedentary controls during the first week in males (Extended Data Fig. 2-2*E*). During Weeks 2–4, male and female runners consumed significantly more calories than their sedentary counterparts (Extended Data Fig. 2-2*E*,*G*). After 28 d, cumulative caloric intake did not differ between male runners and controls but was higher in female runners compared with female controls (Extended Data Fig. 2-2*F*,*H*).

Running also impacted daily water consumption (Extended Data Fig. 2-2*I*–*L*). From Week 2 onward, both male and female runners showed a trend to consume more water than their respective sedentary counterparts; however, post hoc analyses did not reveal significant group differences, and cumulative water intake did not differ between conditions (Extended Data Fig. 2-2*I*–*L*).

After 28 d, terminal gWAT weight was lower in both male and female runners compared with their respective sedentary controls (Extended Data Fig. 2-3*A*,*C*). Moreover, terminal gWAT weight negatively correlated with total running distance in males, whereas no correlation was observed in females (Extended Data Fig. 2-3*B*,*D*). In contrast, adrenal weight normalized to body weight was not significantly affected by running in either sex, and no correlations were observed with total running distance (Extended Data Fig. 2-3*E*–*H*).

In summary, long-term VWR limited body weight gain and lowered adiposity, particularly in males, without significantly altering adrenal weight.

### Long-term VWR modulates regional ΔFOSB in males and females

Next, we evaluated the impact of long-term VWR on ΔFOSB-positive cell numbers in 46 brain regions associated with stress-, cognition-, and reward-related behavior in male and female mice. To do this, we adapted an atlas-based analysis workflow (Bourgeois et al., 2021) for bilateral mouse brain sections and compared these outcomes with those of sedentary controls (Fig. 1*B*,*C*; see Extended Data Figs. 3-1 and 4-1 for a full overview of all included ROIs, Bregma ranges, number of slices included per group, percentage of change in runners, and statistics).

In males, running significantly increased ΔFOSB-positive cell numbers in the dorsomedial striatum (DMS), the intermediate part of the lateral septum (LSI), the dorsal (dGrDG) and ventral (vGrDG) granule cell layers of the dentate gyrus, and the ventral cornu ammonis 2 (vCA2; all *p* < 0.05; Fig. 3*A*,*B*; Extended Data Fig. 3-1). Male runners also showed a trend toward increased ΔFOSB-positive numbers in the cingulate cortex area 2 (Cg2) and the dorsolateral striatum (DLS; both *p* < 0.10; Fig. 3*A*,*B*; Extended Data Fig. 3-1).

**Figure 3.**
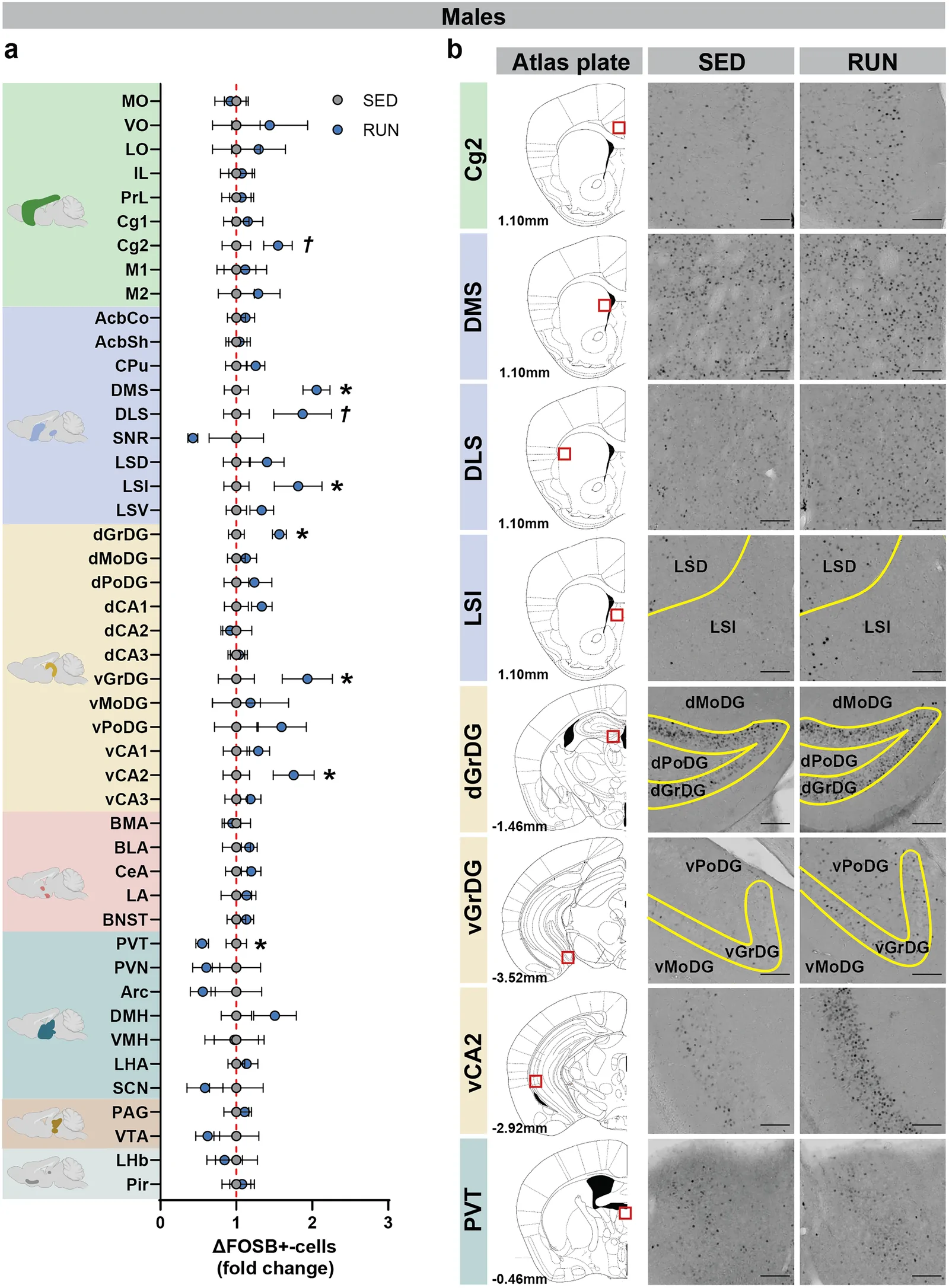
Long-term VWR induction of ΔFOSB in brain regions of individually housed male C57BL/6J^OlaHsd^ mice. ***a***, Fold change in the mean number of ΔFOSB-positive cells (per mm^2^) per ROI in male runners (RUN), normalized to the mean of sedentary (SED) controls; *p < 0.05, ^†^*p* < 0.10 before FDR correction (see Extended Data Fig. 3-1 for explanation of abbreviations and exact statistical values). ***b***, Representative images of ΔFOSB immunoreactivity in the Cg2, DMS, DLS, intermediate part of the lateral septum (LSI), the dorsal (dGrDG), and ventral (vGrDG) part of the granule cell layer of the dentate gyrus, the ventral cornu ammonis 2 (vCA2), and the PVT. Scale bar, 100 µm. Data are presented as the mean ± SEM (***a***). (***a***) *n* = 10–12/group. Detailed statistics and full list of abbreviations are shown in Extended Data Figure 3-1. Pearson’s correlations between total running distance and brain ΔFOSB in males are shown in Extended Data Figure 3-2.

10.1523/ENEURO.0066-26.2026.f3-1 Figure 3-1 **Quantification of ΔFOSB per brain region of interest in individually-housed male C57BL/6J^OlaHsd^ mice**. Fold change in ΔFOSB**-**positive cells between male sedentary (SED) controls and runners (RUN). Data were compared with an independent Student’s t-test (indicated with *t*) or a Mann-Whitney U-test (indicated with *U*) depending on data distribution. *N* SED*/N* RUN = number of mice; *P* = nominal *p­*-value; *P* adjusted = *P*-values after FDR correction for multiple comparisons; *% change* = fold change in ΔFOSB-positive cells in RUN mice compared to SED controls; Bregma range = Bregma ranges included per brain region; *N* slices = range of the min and max number of slices per brain region per group. Significant *P*-values (< 0.05) are marked in bold. Download Figure 3-1, TIF file.

10.1523/ENEURO.0066-26.2026.f3-2 Figure 3-2 **Pearson correlations between total running distance and brain ΔFOSB in individually-housed male C57BL/6J^OlaHsd^ mice**. (**a**) Heatmap of Pearson correlation values (r) of total running distance and fold change in ΔFOSB-positive cells per region of interest (ROI) in male runners (RUN). *†P* < 0.10. See Figure 3-1 for explanation of used brain region abbreviations. (**b**) Correlation between total running distance and mean number of ΔFOSB-positive cells in the ventral molecular layer of the dentate gyrus (vMoDG; R² = 0.346, F_(1,8)_ = 4.233, *P* = 0.074) in male RUN mice. Data are presented as heatmaps indicating Pearson correlation coefficients (r) (a) or as regression plots indicating the simple linear regression (line) with dots representing individual mice (b). (a, b) *n* = 10/group. Download Figure 3-2, TIF file.

In contrast, running significantly decreased ΔFOSB-positive cell numbers in the paraventricular thalamic nucleus (PVT; *p* < 0.05; Fig. 3*A*,*B*; Extended Data Fig. 3-1). After applying FDR correction for multiple comparisons, only the increases observed in the DMS and dGrDG remained statistically significant (Extended Data Fig. 3-1). A trend toward a negative correlation between total running distance and ΔFOSB-positive numbers was observed in the ventral molecular layer of the dentate gyrus (vMoDG; Extended Data Fig. 3-1*A*,*B*), but this correlation did not survive multiple-comparisons correction.

In females, running significantly increased ΔFOSB-positive cell numbers in the medial orbital cortex (MO), Cg2, and prelimbic cortex (PrL; *p* < 0.05), with trends toward an increase in the ventral orbital cortex (VO), Cg1, DMS ventral polymorph layer of the dentate gyrus (vPoDG), and PVT (all *p* < 0.10; Fig. 4*A*,*B*; Extended Data Fig. 4-1). Conversely, running significantly reduced ΔFOSB-positive cell numbers in the dGrDG (Fig. 4*A*,*B*; Extended Data Fig. 4-1). After FDR correction, only the effects in the DMS and dGrDG remained statistically significant (Extended Data Fig. 4-1). Running distance correlated positively with ΔFOSB-positive cell numbers in the dorsal molecular layer of the dentate gyrus (dMoDG) and vCA3 and showed a trend for a positive correlation in the lateral habenula (LHb; Extended Data Fig. 4-1*A*,*E*–*G*). Running distance negatively correlated with ΔFOSB-positive cell numbers in the dorsal part of the lateral septum and showed a trend (*p* < 0.10) for negative correlations with ΔFOSB-positive cell numbers in the MO and VO (Extended Data Fig. 4-1*A*–*D*). However, none of these (significant) correlations survived FDR correction for multiple comparisons.

**Figure 4.**
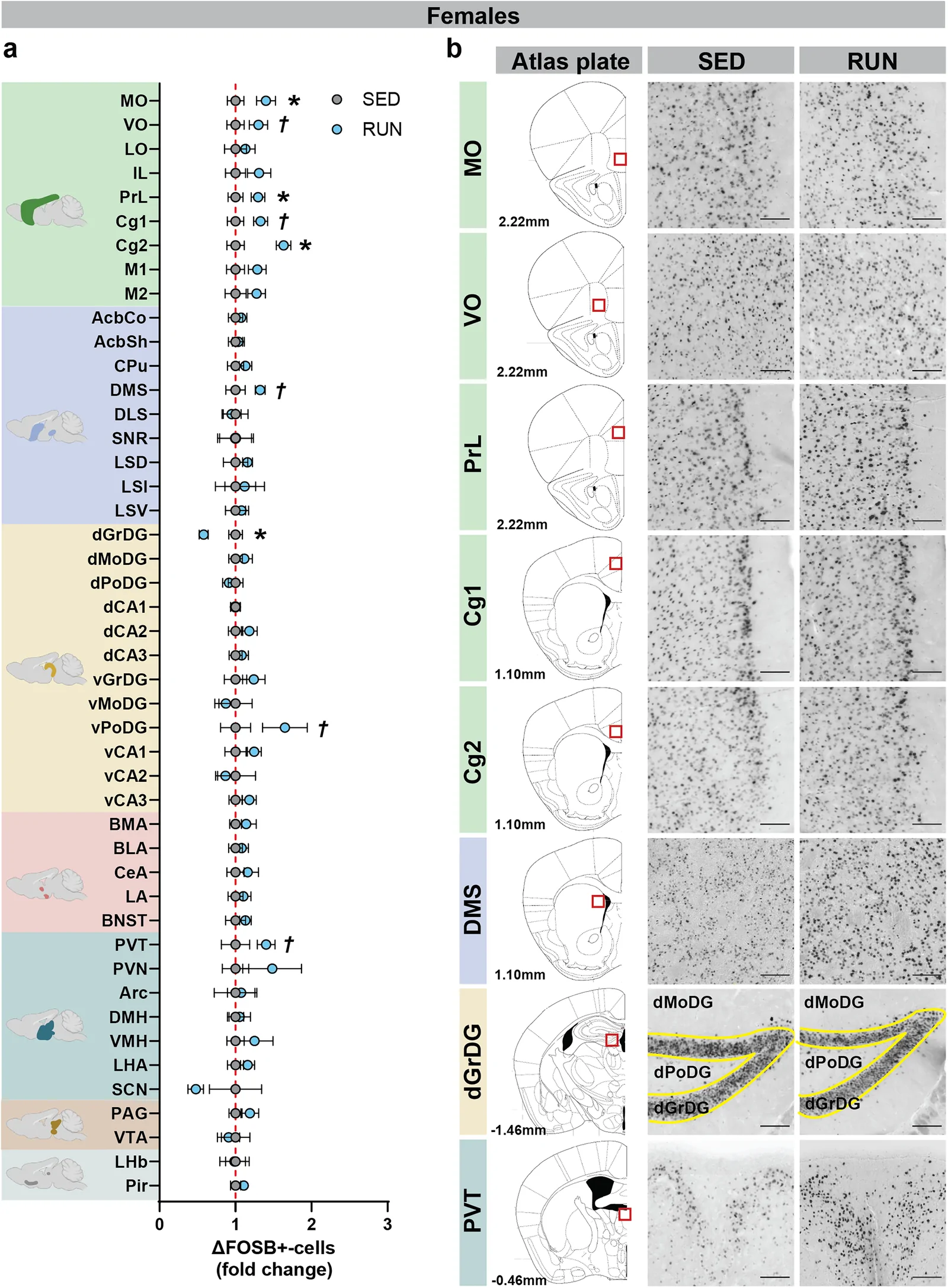
Long-term VWR induction of ΔFOSB in brain regions of individually housed female C57BL/6J^OlaHsd^ mice. ***a***, Fold change in the mean number of ΔFOSB-positive cells (per mm^2^) per ROI in female runners (RUN), normalized to the mean of sedentary (SED) controls; *p < 0.05, ^†^*p* < 0.10 before FDR correction (see Extended Data Fig. 4-1 for the explanation of abbreviations and exact statistical values). ***b***, Representative images of ΔFOSB immunoreactivity in the medial (MO) and ventral orbital cortex (VO), prelimbic cortex (PrL), cingulate cortex 1 (Cg1) and 2 (Cg2), DMS, dorsal part of the granule cell layer of the dentate gyrus (dGrDG), and the PVT. Scale bar, 100 µm. Data are presented as the mean ± SEM (***a***). (***a***) *n* = 12/group. Detailed statistics and full list of abbreviations are shown in Extended Data Figure 4-1. Pearson’s correlations between total running distance and brain ΔFOSB in males are shown in Extended Data Figure 4-2.

10.1523/ENEURO.0066-26.2026.f4-1 Figure 4-1 **Quantification of ΔFOSB per brain region of interest in individually-housed female C57BL/6J^OlaHsd^ mice**. Fold change in ΔFOSB**-**positive cells between female sedentary (SED) controls and runners (RUN). Data were compared with an independent Student’s t-test (indicated with *t*) or a Mann-Whitney U-test (indicated with *U*) depending on data distribution. *N* SED*/N* RUN = number of mice; *P* = nominal *p­*-value; *P* adjusted = *P*-values after FDR correction for multiple comparisons; *% change* = fold change in ΔFOSB-positive cells in RUN mice compared to SED controls; Bregma range = Bregma ranges included per brain region; *N* slices = range of the min and max number of slices per brain region per group. Significant *P*-values (< 0.05) are marked in bold. Download Figure 4-1, TIF file.

10.1523/ENEURO.0066-26.2026.f4-2 Figure 4-2 **Pearson correlations between total running distance and brain ΔFOSB in individually-housed female C57BL/6J^OlaHsd^ mice.** (**a**) Heatmap of Pearson correlation values (r) of total running distance and fold change in ΔFOSB-positive cells per region of interest (ROI) in female runners (RUN; *P < 0.05, *†P* < 0.10). See Figure 4-1 for explanation of used brain region abbreviations. (**b**-**g**) Correlation between total running distance and mean number of ΔFOSB-positive cells in (b) medial (MO; R² = 0.274, F_(1,9)_ = 3.403, *P* = 0.098) and (c) ventral orbital cortex (VO; R² = 0.281, F_(1,9)_ = 3.523, *P* = 0.093), (d) dorsal part of the lateral septum (LSD; R² = 0.424, F_(1,9)_ = 6.612, *P* = 0.030), (e) dorsal part of the molecular layer of the dentate gyrus (dMoDG; R² = 0.384, F_(1,9)_ = 5.598, *P* = 0.042), (f) ventral part of the cornu ammonis 3 (vCA3; R² = 0.417, F_(1,9)_ = 6.427, *P* = 0.032) and (g) lateral habenula (LHb; R² = 0.330, F_(1,9)_ = 4.428, *P* = 0.065) in female RUN mice. Data are presented as heatmaps indicating Pearson correlation coefficients (r) (a) or as regression plots indicating the simple linear regression (line) with dots representing individual mice (b-g). (a-g) *n* = 11/group. Download Figure 4-2, TIF file.

Taken together, these observations demonstrate that long-term VWR impacts ΔFOSB immunoreactivity in several brain regions involved in stress-, cognition-, and reward-related behavior in males and females.

### Long-term VWR alters brain ΔFOSB coactivation network efficiency and topology in males

To further explore how long-term VWR influenced ΔFOSB immunoreactivity at the network level, we analyzed ΔFOSB coactivation patterns by cross-correlating regional ΔFOSB immunoreactivity in male and female sedentary controls and runners. For further network characterization, we quantified global (i.e., density, small-worldness) and local (i.e., degree, betweenness centrality) network metrics.

Sedentary males exhibited a densely interconnected network, as shown by both the coactivation heatmap and network visualizations, with multiple regions across distinct structures occupying central positions (Fig. 5*A*,*B*). Consistent with this pattern, network density was relatively high in sedentary controls (*k_DEN_* = 0.278; Fig. 5*C*). In contrast, male runners showed an overall modest reduction in connectivity (*k_DEN_* = 0.251; Fig. 5*C*), accompanied by a pronounced shift toward greater small-world-like organization (SED, *σ* = 2.294; RUN, *σ* = 2.793; Fig. 5*D*). As a higher small-worldness coefficient reflects greater clustering (transitivity) and shorter average path lengths, this observed increase in small-world-like properties suggests enhanced global network efficiency. These group differences were robust across multiple correlation thresholds (*α* = 0.05, 0.01, 0.0001; Extended Data Fig. 5-1*A*,*B*), confirming that VWR reliably alters network organization in males.

**Figure 5.**
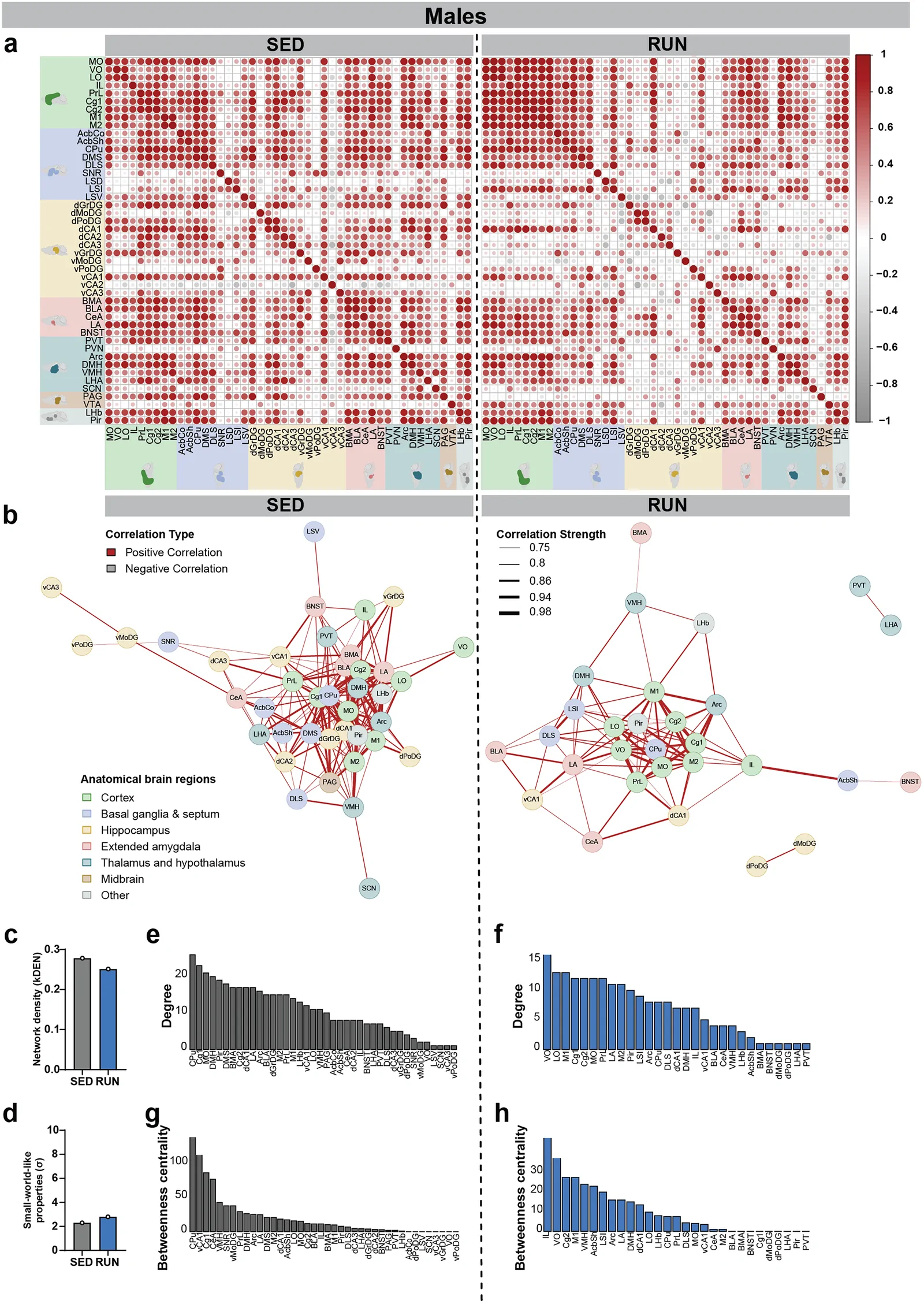
Network analysis reveals altered brain ΔFOSB coactivation following long-term VWR in individually housed male C57BL/6J^OlaHsd^ mice. ***a***, Unweighted regional cross-correlation matrices of ΔFOSB-positive cells per square millimeter between all pairs of neuroanatomical brain regions of male sedentary controls (SED) and runners (RUN). ***b***, ΔFOSB coactivation networks constructed after thresholding for the strongest and most significant correlated or anticorrelated connections (*r* > 0.7, *p* < 0.005) in SED and RUN mice. ***c***, Network density expressed as *k_DEN_* of SED and RUN mice. ***d***, Small-world-like properties expressed as *σ* of SED and RUN mice. ***e*, *f***, Node degree per ROI of (***e***) SED and (***f***) RUN mice. ***g*, *h***, Betweenness centrality per ROI of (***g***) SED and (***h***) RUN mice. (***a*–*h***) *n* = 10–12/group. (***b*–*h***) *p* < 0.005 was considered significant. The influence of thresholding on network topology in males is shown in Extended Data Figure 5-1.

10.1523/ENEURO.0066-26.2026.f5-1 Figure 5-1 **The influence of thresholding on network topology in individually-housed male C57BL/6J^OlaHsd^ mice.** (**a**) Regional cross-correlation matrices of ΔFOSB-positive cells between all pairs of neuroanatomical regions in male sedentary (SED) controls and runners (RUN), at thresholds of *α* < 0.05, *α* < 0.01 and *α* < 0.0001. (**b**) Functional connectivity networks constructed for SED and RUN mice, at thresholds of *α* < 0.05, *α* < 0.01 and *α* < 0.0001. (a, b) *n* = 10-12/group. Download Figure 5-1, TIF file.

Local network metrics further provided insight into the structural shift observed at the whole-network level. Both male runners and controls showed a long-tailed distribution of degree and betweenness centrality, reflecting the presence of strongly influential hub nodes (Fig. 5*E*–*H*). In sedentary males, high-degree nodes were predominantly located in the striatum [caudate putamen (CPu)], cortex (Cg1, MO), hypothalamus [dorsomedial hypothalamus (DMH)], and piriform cortex (Pir), consistent with the broad, multisystem centrality observed in the network visualizations (Fig. 5*B*,*E*). Betweenness centrality analysis supported this architecture, identifying striatal (CPu), hippocampal (vCA1), cortical (Cg1), amygdalar [central amygdala (CeA)], and hypothalamic [ventromedial hypothalamus (VMH)] as dominant hubs (Fig. 5*G*). In male runners, both degree and betweenness shifted prominently toward cortical regions—including VO, LO, M1, Cg1, Cg2, MO, PrL, and infralimbic cortex (IL)—reflecting the cortico-centric architecture also identified in the coactivation maps (Fig. 5*F*,*H*).

Taken together, these results demonstrate that long-term VWR fundamentally reshapes ΔFOSB coactivation networks in males by lowering overall connectivity, improving network efficiency, and reallocating hub status toward cortical systems. This suggests a reorganization that favors a more streamlined, cortical-centered information flow while reducing diffuse connectivity.

### Long-term VWR alters ΔFOSB coactivation network efficiency and topology in females

In sedentary females, coactivation heatmaps and network plots revealed two smaller networks: one primarily involving the prefrontal cortex, dorsal striatum, and Pir and the other primarily involving the hippocampus, amygdala, and lateral hypothalamus, interconnected via the bed nucleus of the stria terminalis (BNST; Fig. 6*A*,*B*). The presence of these two partially segregated subnetworks is relevant for the interpretation of local network metrics, as measures such as degree and betweenness centrality may be distributed across subnetworks rather than concentrated within a single integrated network.

**Figure 6.**
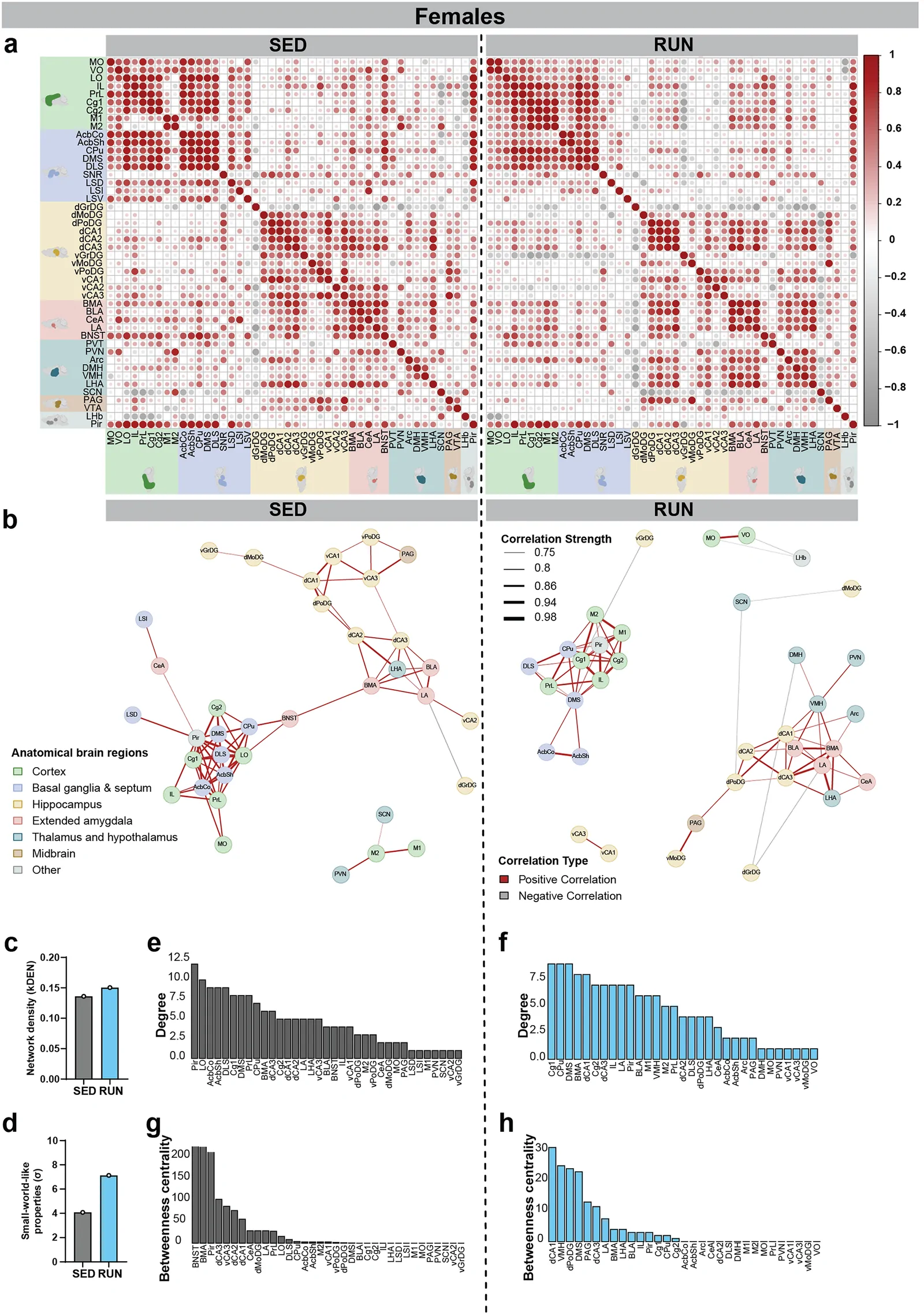
Network analysis reveals altered brain ΔFOSB coactivation following long-term VWR in individually housed female C57BL/6J^OlaHsd^ mice. ***a***, Unweighted regional cross-correlation matrices of ΔFOSB-positive cells per square millimeter between all pairs of neuroanatomical brain regions of female sedentary controls (SED) and runners (RUN). ***b***, ΔFOSB coactivation networks constructed after thresholding for the strongest and most significant correlated or anticorrelated connections (*r* > 0.7, *p* < 0.005) in SED and RUN mice. ***c***, Network density expressed as *k_DEN_* of SED and RUN mice. ***d***, Small-world-like properties expressed as *σ* of SED and RUN mice. ***e*, *f***, Node degree per ROI of (***e***) SED and (***f***) RUN mice. ***g*, *h***, Betweenness centrality per ROI of (***g***) SED and (***h***) RUN mice. ***a*–*h***, *n* = 10–12/group. ***b*–*h***, *p* < 0.005 was considered significant. The influence of thresholding on network topology in females is shown in Extended Data Figure 6-1.

10.1523/ENEURO.0066-26.2026.f6-1 Figure 6-1 **The influence of thresholding on network topology in individually-housed female C57BL/6J^OlaHsd^ mice.** (**a**) Regional cross-correlation matrices of ΔFOSB-positive cells between all pairs of neuroanatomical brain regions in female sedentary (SED) controls and runners (RUN), at thresholds of *α* < 0.05, *α* < 0.01 and *α* < 0.0001. (**b**) Functional connectivity networks constructed for SED and RUN mice, at thresholds of *α* < 0.05, *α* < 0.01 and *α* < 0.0001. (a, b) *n* = 12/group. Download Figure 6-1, TIF file.

In female runners, coactivation networks largely resembled those of their sedentary counterparts but showed subtle differences, including greater involvement of motor areas and reduced centrality of the orbital cortex, nucleus accumbens (NAc), and ventral hippocampal regions (Fig. 6*B*). Additionally, dorsal hippocampal and amygdala regions exhibited stronger connections with hypothalamic areas but weaker connections with the lateral hypothalamus (Fig. 6*B*). Network density was lower in sedentary females compared with sedentary males (*k_DEN_* = 0.136; Fig. 6*C*), whereas female runners showed a modest increase in connectivity (*k_DEN_* = 0.151; Fig. 6*C*). These changes were accompanied by a marked increase in small-worldness (SED, *σ* = 4.075; RUN, *σ* = 7.127; Fig. 6*D*) and were consistent across multiple thresholds (*α* = 0.05, 0.01, 0.0001; Extended Data Fig. 6-1*A*,*B*). At the most stringent threshold (*p* < 0.0001), connections were largely confined to cortical regions, indicating that within-cortex connectivity represented the strongest network interactions in female runners (Extended Data Fig. 6-1*A*,*B*).

Local network metrics further highlighted subtle structural shifts. Both running and sedentary females displayed a long-tailed distribution of degree and betweenness centrality, reflecting strongly influential hub nodes (Fig. 6*E*–*H*). In sedentary females, high-degree nodes were primarily cortical (LO), striatal [NAc core (AcbCo), NAc shell (AcbSh), DLS], or the Pir (Fig. 6*B*,*E*), aligning with one of the subnetworks observed (Fig. 6*A*,*B*). Remarkably, betweenness centrality analysis identified hubs corresponding to the second network, including (extended) amygdalar [BNST, basomedial amygdala (BMA)] and hippocampal (dCA3, vCA3, dCA2, dCA1) areas (Fig. 6*A*,*B*,*G*). In female runners, degree and betweenness shifted slightly but still highlighted striatal (CPu, DMS), amygdala (BMA), and hippocampal [dCA1 and dorsal polymorph layer of the dentate gyrus (dPoDG)] regions as main hubs, while the VMH and periaqueductal gray (PAG) emerged as new central nodes (Fig. 6*F*,*H*).

Taken together, these results demonstrate that, also in females, VWR fundamentally reshapes ΔFOSB coactivation networks by increasing global efficiency while generally maintaining network topology.

### Permutation analyses reveal specific connections and broad connection patterns impacted by long-term VWR and sex

Next, we applied permutation testing to compare correlation matrices between experimental groups and to identify individual edges showing significant changes between groups. To visualize overall patterns of change, we first computed an unthresholded difference matrix by subtracting correlation coefficients of sedentary males from those of running males and displayed these values as a heatmap (Fig. 7*A*, left). This revealed stronger correlations among cortical regions, as well as between cortical regions with other brain regions, such as striatal regions and ventral hippocampal regions, in male runners (Fig. 7*A*, left). Furthermore, we observed a pattern of reduced correlations between mainly dorsal hippocampal regions and other brain regions, such as cortical regions and striatal regions (Fig. 7*A*, left). In addition, several correlations with the amygdala, ventral hippocampal, and (hypo)thalamic regions, as well as with the ventral tegmental area (VTA), were stronger in male runners (Fig. 7*A*, left).

**Figure 7.**
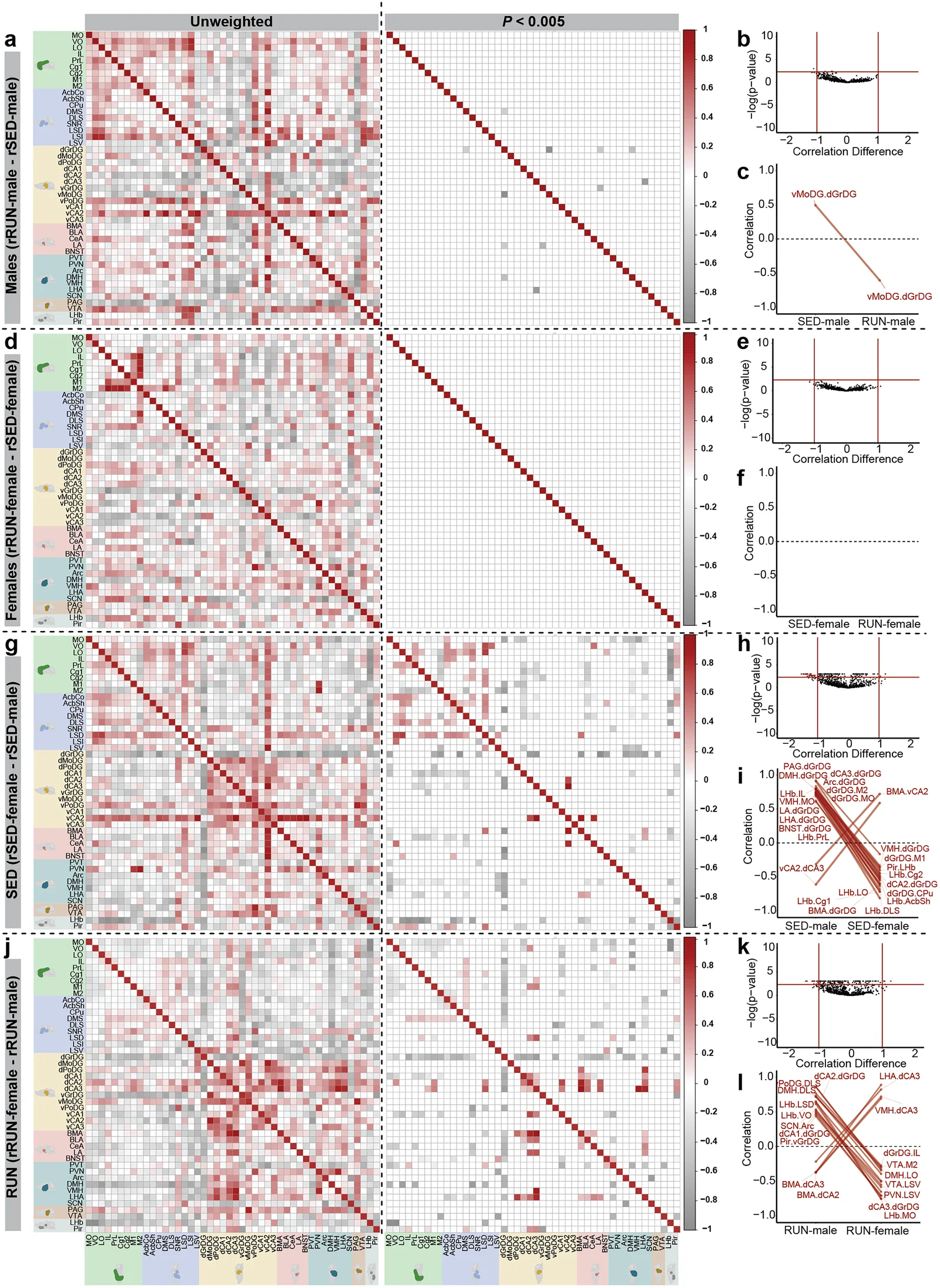
Permutation analysis reveals sex-specific long-term VWR-mediated alterations in brain ΔFOSB coactivation networks. ***a*, *d*, *g*, *j***, Unweighted and weighted (*p* < 0.005) heatmaps of Pearson’s correlation differences (*r*_group2_ − *r*_group1_) for all individual regional connections calculated from permutation testing. The Mantel test revealed significant overall differences between the unweighted heatmaps of (***a***) sedentary (SED) and running (RUN) males (Mantel's *z* = 225.168, *p* = 0.001; ***d***) and between SED and RUN females (Mantel's *z* = 141.206, *p* = 0.001), (***j***) as well as between male and female RUN mice (Mantel's *z* = 198.995, *p* = 0.001), (***g***) but not male and female SED controls (Mantel's *z* = 129.700, *p* = 0.135). ***b*, *e*, *h*, *k***, Volcano plots of Pearson’s correlation differences (*r*_group2_ − *r*_group1_) for all individual regional connections against their *p* values calculated from a permutation analysis. Points intersecting or within the top-left or top-right quadrant represent the regional relationships with the greatest change (|correlation difference| > 1) that were most significant. ***c*, *f*, *i*, *I***, Parallel coordinate plots highlighting individual significantly changed regional correlations between groups. (***a*–*l***) *n* = 9–12/group. (***a*–*l***) *p* < 0.005 was considered significant.

To determine whether these changes reflected a reorganization of global network structure, we compared the full coactivation matrices using a Mantel test, which revealed a significant difference in overall coactivation matrices between male runners and sedentary controls (Fig. 7*A*, left). Next, to reveal local changes in the network, we performed permutation testing on individual correlations using a stringent threshold (*p* < 0.005) and identified significantly reduced correlations involving several hippocampal nodes [i.e., dGrDG–DMS, dGrDG–vMoDG, dGrDG–LA, dGrDG–DMH, dCA3–lateral hypothalamus (LHA), and vGrDG–LA; Fig. 7*A*, right]. To detect correlations that reversed direction between groups, we applied an additional criterion [*p* < 0.005; *r*(difference) > 1], which revealed that VWR produced a shift in the relationship between the vMoDG and the dGrDG, converting a positive correlation in male sedentary controls into a negative correlation in male runners (Fig. 7*B*,*C*). Collectively, these findings indicate that VWR in males produces both global and local shifts in the coactivation networks of male mice.

In females, visualization of unthresholded difference matrices revealed more spatially dispersed alterations in correlations between sedentary and running mice (Fig. 7*D*, left). Despite this distributed pattern, comparison of the full coactivation matrices using a Mantel test indicated a significant difference in overall coactivation structure between female runners and sedentary controls (Fig. 7*D*, left). However, permutation testing of individual interregional correlations using the same stringent criteria [*p* < 0.005; with or without *r*(difference) > 1] did not identify any specific connections that differed significantly between groups (Fig. 7*D*, right, *E*,*F*), suggesting that VWR-related changes in females were broadly distributed across the network rather than driven by large shifts in specific correlations.

To evaluate baseline sex differences, we visualized unthresholded correlation differences by subtracting values of sedentary males from those of sedentary females (Fig. 7*G*, left). This comparison revealed stronger correlations in sedentary females among cortical, striatal, ventral hippocampal, and amygdala regions, accompanied by reduced correlations involving dorsal hippocampal regions. To determine whether these changes reflected differences in global network organization, we compared full coactivation matrices using a Mantel test. Despite region-specific alterations, overall interregional correlation structure did not differ significantly between sedentary males and females (Fig. 7*G*, left).

To identify specific connections underlying these local differences, permutation testing was applied using stringent criteria [*p* < 0.005; *r*(difference) > 1]. The most pronounced downregulated connections in sedentary females involved the dGrDG, which showed reduced correlations with amygdala [BNST, BMA, lateral amygdala (LA)], hypothalamic [LHA, DMH, VMH, arcuate nucleus (Arc)], (motor) cortical (MO, M1, M2), hippocampal (dCA2, dCA3), striatal (CPu), and midbrain (PAG) regions (Fig. 7*H*,*I*). In addition, LHb connectivity with cortical [lateral orbital cortex (LO), PrL, IL, Cg2, Cg1], striatal (AcbSh, DLS), and Pir areas was reduced, as was connectivity between the VMH and MO. Conversely, correlations involving the vCA2 with dCA3 and BMA were increased in sedentary females compared with sedentary males (Fig. 7*H*,*I*). Together, these findings indicate localized sex differences in specific interregional correlations at baseline without a corresponding shift in global network architecture.

Finally, we examined sex differences following VWR. Visualization of unthresholded difference matrices comparing male and female runners revealed stronger hippocampal connectivity in females, excluding connections involving dGrDG and dMoDG. Enhanced correlations were observed both within hippocampal circuits and between hippocampal, amygdala, and hypothalamic regions, whereas cortical connectivity showed comparatively minor differences (Fig. 7*J*, left).

Statistical comparison of the full coactivation matrices via a Mantel test revealed that there is a significant difference in overall network organization between male and female runners (Fig. 7*J*, left), indicating sex-dependent reorganization of network architecture following VWR. Permutation-based edge-level analyses identified multiple changes in female runners. Reduced correlations were observed between dGrDG and hippocampal (dCA1, dCA2, dCA3) and cortical (IL) regions, as well as between vGrDG and Pir, and between vPoDG and DLS (Fig. 7*K*,*L*). Additional reductions involved LHb connectivity with MO, VO, and dorsal lateral septum (LSD), as well as several hypothalamic and midbrain connections, including suprachiasmatic nucleus (SCN)–Arc, DMH–DLS, DMH–LO, paraventricular hypothalamic nucleus (PVN)–ventral lateral septum (LSV), VTA–LSV, and VTA–M2. Conversely, female runners exhibited increased correlations between amygdala and hippocampal regions (BMA–dCA2, BMA–dCA3) and between hypothalamic and hippocampal regions (LHA–dCA3, VMH–dCA3) relative to male runners (Fig. 7*K*,*L*).

Together, these findings indicate that VWR induces sex-specific reorganization of ΔFOSB coactivation patterns.

## Discussion

Here, we demonstrate that long-term VWR in individually housed male and female C57BL/6J^OlaHsd^ mice resulted in differences in ΔFOSB immunoreactivity in several brain regions previously implicated in stress regulation and cognition- and reward-related behavior. Network analyses revealed that VWR reorganized brain ΔFOSB coactivation networks compared with sedentary controls, in distinct ways across sexes. Indeed, male runners showed marked topological reorganization toward a more cortico-central network architecture, accompanied by relatively modest changes in network density and global efficiency, whereas female runners exhibited fewer alterations in network topology, but a more pronounced increase in global efficiency.

### VWR-mediated changes in regional ΔFOSB immunoreactivity

This study provides a first characterization of VWR-mediated ΔFOSB modulation in the female mouse brain, revealing, among others, sustained activation of cortical regions. In addition, we identify VWR-mediated modulation of ΔFOSB in several novel brain regions in male runners, including the lateral septum and PVT, extending beyond previously reported VWR modulation of ΔFOSB in striatal and hippocampal structures in male rats and mice (Nishijima et al., 2013; Herrera et al., 2016; Mul et al., 2018).

Some of these modulations appear to be different in males and females. For example, VWR decreased PVT ΔFOSB in males but trended to increase PVT ΔFOSB in females, all relative to controls. To the best of our knowledge, a significant decrease in ΔFOSB or trending for a significant decrease, following long-term VWR, has to date been reported for a few brain regions: the SCN in male and female running rats (Shiba et al., 2025), the arcuate nucleus in female running rats (Hardonk et al., 2026), and the basolateral amygdala in female running prairie voles (Watanasriyakul et al., 2019). Given the established role of the PVT in stress responsiveness, arousal, and regulation of mesolimbic dopamine signaling (Ren et al., 2018; Elam et al., 2025), our observation may reflect sex-specific recruitment of this stress-sensitive hub during long-term VWR. Because chronic stress induces ΔFOSB accumulation in PVT neurons and suppression of these neurons produces antidepressant-like effects in male, but not female, mice (Zhao et al., 2021), this suggests that decreased ΔFOSB in the male PVT could be relevant for stress-related adaptations to VWR; however, this study did not directly assess stress-related behavior, and therefore the functional significance of our observations remains to be determined.

We also observed VWR-mediated suppression of ΔFOSB in the dGrDG of female mice. Whereas prior studies in female rats and prairie voles reported no VWR-induced modulation of dorsal hippocampal ΔFOSB (Watanasriyakul et al., 2019; Hardonk et al., 2026), the present findings suggest a sex-specific adaptation of the dentate gyrus to VWR. In male mice and rats, VWR seems to consistently increase dorsal hippocampal (DG) and ventral hippocampal (DG, CA1, CA3) ΔFOSB (Nishijima et al., 2013; Hardonk et al., 2026). In contrast, the present data indicate that long-term VWR results in reduced ΔFOSB accumulation in the female dGrDG. However, the opposing ΔFOSB changes between sexes may reflect sex-dependent routes toward circuit adaptations during long-term VWR rather than opposing effects on hippocampal function. Future studies should assess the underlying mechanisms that drive the sex-dependent VWR modulation of dGrDG ΔFOSB and its subsequent functional implication.

Our large-scale ΔFOSB brain signatures following long-term VWR in mice extend our prior work in rats (Hardonk et al., 2026), and several patterns of VWR-induced ΔFOSB modulation were consistent between the mouse and rat study. For example, dorsal striatal, hippocampal, and cingulate regions showed consistent induction in male rats and mice, whereas cortical and ventral hippocampal regions were predominantly affected in female rats and mice. These observations suggest that core networks are engaged by VWR in both mice and rats.

Nonetheless, the number of brain regions showing greater VWR-mediated ΔFOSB immunoreactivity, compared with sedentary controls, appeared lower in mice than in rats, with the latter showing effects in the medial prefrontal areas, AcbSh, multiple ventral dentate gyrus subregions, and select amygdaloid and hypothalamic nuclei (Hardonk et al., 2026). Notably, ΔFOSB accumulation in the NAc, consistently reported in previous mouse and rat VWR studies (Werme et al., 2002; Greenwood et al., 2011; Obici et al., 2015; Herrera et al., 2016; Mul et al., 2018), was not observed in the current mouse study. However, an important methodological difference is that our study used light, low-resistance horizontal (saucer-like) plastic running wheels instead of the heavier, higher-resistance stainless-steel vertical wheels employed in most mouse and rat studies to date (Werme et al., 2002; Greenwood et al., 2011; Obici et al., 2015; Herrera et al., 2016; Mul et al., 2018). Differences in physical demand when running on these two wheel designs may differentially impact recruitment of effort- and reward-related circuits to sustain motivation for running, thereby possibly contributing to differences in ΔFOSB levels in limbic and motivational regions.

Taken together, our findings indicate that VWR engages conserved core networks in both rats and mice but also suggest that both species and wheel design are factors that impact neural circuit recruitment, highlighting the need for direct comparisons between wheel designs within species in future studies.

### VWR effects on ΔFOSB coactivation networks

As brain regions do not operate in isolation and ΔFOSB reflects patterns of sustained neural activity (McClung et al., 2004), we also examined ΔFOSB coactivation networks. We previously observed that long-term VWR in both male and female rats produced more efficient networks with reduced density and cortical-favored information flow (Hardonk et al., 2026). In the present study, VWR promoted a similar pattern in males, with an even stronger reorganization toward a cortex-centered architecture, accompanied by modest reductions in density and increased global efficiency. In females, VWR robustly increased global efficiency, consistent with prior rat data, but lacked density reduction or pronounced shifts toward cortical centrality. One possible explanation is that VWR induced the emergence of two distinct coactivation networks in female runners rather than a single integrated network. This may complicate the interpretation of local network characteristics to describe network centrality (i.e., node degree and betweenness centrality).

Notably, cortical regions constituted one of these networks, and applying more stringent thresholds (*p* < 0.0001) revealed that these within-cortex connections were stronger than the central nodes identified using a threshold of *p* < 0.005. An alternative, and not mutually exclusive, explanation is that sedentary females already display a more cortex-centered network organization at baseline, characterized by stronger intracortical connectivity and lower overall network density. To test this, we directly compared ΔFOSB coactivation networks between sedentary males and females. Indeed, female sedentary animals showed higher cortical connections than sedentary males, together with fewer connections linking the dGrDG, amygdala, and hypothalamic structures, as well as reduced connectivity involving the LHb. After VWR, this female-specific cortical predominance was diminished, indicating that females start from a more cortex-centered, lower-density network configuration that may mask further VWR-induced cortical reorganization.

### Methodological considerations and limitations

Several limitations to the present study should be noted. First, male brain sections yielded robust ΔFOSB immunoreactivity signals, whereas female brain sections required antigen retrieval to obtain similar levels. Although this may have influenced absolute ΔFOSB levels, our main conclusions regarding effects in males and females are based on within-animal patterns of regional ΔFOSB coactivation and graph-theoretical metrics derived from the covariance structure across brain regions rather than on absolute signal intensity. Antigen retrieval in females is expected to act as a global sensitivity enhancement across regions and therefore should not selectively create or abolish the correlation structure that underlies our network-level analyses. While we agree that the difference in staining protocol warrants caution when directly interpreting absolute male–female differences in ΔFOSB density, we consider it unlikely that this factor alone accounts for the observed differences in network topology that we report in male and female runners.

Second, the antibody used here recognizes FOSB, vFOSB, ΔFOSB, and Δ2ΔFOSB (Ohnishi et al., 2011). Therefore, wheels were blocked for 24 h prior to brain tissue collection to allow degradation of the short-lived full-length FOSB and vFOSB proteins, such that any remaining immunoreactivity reflects ΔFOSB (Nestler et al., 2001; Perrotti et al., 2004). However, we cannot fully exclude that a small amount of immunoreactivity reflects FOSB, vFOSB, or Δ2ΔFOSB.

Third, estrous cycle stage was not monitored in the female cohort, despite evidence that ovarian hormones, particularly estrogen, can modulate ΔFOSB signaling and related chromatin remodeling across brain regions, at least in sedentary rodents (Rocks et al., 2023; Shiba et al., 2025). Thus, we cannot exclude that some of the observed female-specific patterns may be mediated by hormonal fluctuations, in addition to VWR-mediated effects.

A final limitation is that all mice were housed individually, albeit in open cages which allow for transmission of olfactory and acoustic information. Nonetheless, individual housing may have affected baseline ΔFOSB levels, as solitary housing alters neural activity in stress-related brain regions such as the hippocampus and medial prefrontal cortex, as well as neuronal and behavioral responsivity to chronic social defeat stressors (Wallace et al., 2009; Vialou et al., 2010b; Noback et al., 2021). Because male and female mice from different C57BL/6J substrains demonstrate different metabolic, hormonal, and brain adaptations to short- or long-term social isolation in (early) adulthood, we cannot exclude that this factor influenced the observations in this study (Senst et al., 2016; Rivera-Irizarry et al., 2020; Cepeda et al., 2026; Umamichi et al., 2026).

### Implications and future mechanistic studies

Our large-scale ΔFOSB brain signatures following long-term VWR in mice offer a framework for future mechanistic studies into the impact of VWR-mediated changes in ΔFOSB in brain regions associated with stress regulation and cognition- and reward-related behavior. For example, prior studies in male mice demonstrated that stress- or VWR-induced ΔFOSB accumulation in the NAc (Vialou et al., 2010a,b; Mul et al., 2018), as well as stress induction of ΔFOSB in ventral hippocampus-to-NAc–projecting neurons, promoted stress resilience (Eagle et al., 2020), whereas stress-induced ΔFOSB in the PrL decreased stress resilience (Vialou et al., 2014).

While we did not assess stress-related behavior in the present study, we identify several new regions previously not associated with VWR-mediated ΔFOSB modulation. These included the cingulate cortex and PVT (both sexes), the lateral septum (males), and the orbital cortex (females). Our study thus identifies novel brain regions that might be involved in the effects of VWR on stress regulation and cognition- and reward-related behavior. Additional studies which, e.g., specifically manipulate ΔFOSB in the abovementioned newly identified brain regions and/or study the functional properties of their neurons, will be required to clarify their interdependence and/or unique roles in the VWR-mediated changes in stress regulation and cognition- and reward-related behavior.

In addition to identifying novel candidate brain regions modulated by VWR, our findings further revealed differences in both regional ΔFOSB immunoreactivity and brain network organization within a sex. These differences may shape how exercise-induced neural adaptations relate to responses to chronic stressors, which is relevant for the prevention of depression. Given the higher prevalence of depression in women (Salk et al., 2017; Li et al., 2022) and the historical underrepresentation of females in mechanistic animal studies (Shansky, 2019), our findings highlight the importance of including sex as a biological variable in future mechanistic studies of VWR and its effects on stress regulation-related circuits.

## Conclusion

Taken together, this study demonstrates that long-term VWR in mice modulated ΔFOSB immunoreactivity in brain regions implicated in stress regulation and cognition- and reward-related behavior and reorganized ΔFOSB coactivation networks in distinct ways across sexes. Our large-scale ΔFOSB brain signatures and changes in coactivation networks following long-term VWR in mice provide a framework for functional and mechanistic studies into ΔFOSB-mediated changes in stress regulation and cognition- and reward-related behavior following VWR.

## Data Availability

Access to the data and computational tools used in this study can be obtained from the corresponding author upon reasonable request.

## Synthesis

Reviewing Editor: Adrien Peyrache, McGill University Decisions are customarily a result of the Reviewing Editor and the peer reviewers coming together and discussing their recommendations until a consensus is reached. When revisions are invited, a fact-based synthesis statement explaining their decision and outlining what is needed to prepare a revision will be listed below. The following reviewer(s) agreed to reveal their identity: Jonathan Epp, Balázs Gaszner. Note: If this manuscript was transferred from JNeurosci and a decision was made to accept the manuscript without peer review, a synthesis may not be available.

This manuscript presents a network and graph theoretical analysis of male and female mice response to voluntary exercise. The authors use dFOSB labeling across multiple brain regions to infer chronic or cumulative activation within each region. Running resulted in a number of regions with altered activity in both female and male mice and, at the global level, induced sex specific changes in network properties. The manuscript was evaluated by two reviewers who comments are summarized below.

1. The manuscript focuses on stress reactive brain regions and makes statements along the lines of identifying pathways to target to increase stress resilience. However, in the end the manuscript doesn't examine the impact of stress on these networks or show that modulation of any of the impacted brain regions would have an impact on stress related behaviors. The links to stress and brain resilience should be toned down and the main claims should focus on the impacts of exercise, which is what is actually demonstrated here.

2. The study used immunolabeling by FOSB, yet the antibody controls supporting the specificity of the serum do not seem to be provided. Further details are needed, even perhaps additional controls if possible (e.g. pre-absorption tests, Western blot, or FOSB IHC in combination with in situ hybridization for Fosb mRNA, eventually tests on knockout tissue).

3. It is claimed that the antigen retrieval was required in the female tissues. This questions the comparability of data: on the one hand, this methodological difference may have masked important sex differences in FOSB activity because the weak female FOSB signal was enhanced by epitope retrieval. It is also possible, that the epitope retrieval increased the magnitude of differences unproportionally in females, and they appear now as real differences, that could exist in males also, but we miss them because they were not magnified by the epitope retrieval. It is unclear why the tissues were differently treated. In particular, why was the epitope retrieval not performed on samples of male mice also? This would have made the male-female comparisons reliable.

4. Considering that several brain areas show a very strong sex hormone sensitivity that is in females strongly estrus cycle phase-dependent, it would be informative to know in what cycle phase were the brains collected, if this is known. Otherwise, it should be stated as an limitation of the study, and be discussed.

5. The term brain "map" is used a couple of times. While examining 46 areas is indeed an impressive work, it is still not a brain map yet. For instance, the lower brainstem and cerebellum contain also several mood regulation and motor control-related areas that might have been of interest. Claiming that these data form a brain map is an over interpretation (e.g. see page 23 last paragraph and also elsewhere) and should be toned down.

Minor point:

The term "expression" is used several times. It is better to say immunoreactivity, immunosignal, abundance of FOSB protein, neuronal activity and other synonyms because genes (or mRNAs) are expressed but not the proteins, and this is a protein level study.

## Author Response

To Dr. Adrien Peyrache Reviewing editor of eNeuro Amsterdam, June 12, 2026 Dear Dr. Peyrache, We thank you and the reviewers for the time and effort put into reviewing our manuscript #eN-TNWR-0066-26X and for your positive response and the constructive suggestions, that have further improved our paper. We appreciate the opportunity to submit a revision very much.

Please find attached our revised version, now entitled; 'Exercise training modulates brain ΔFOSB and alters co-activation networks in male and female mice' (#eN-TNWR-0066-26X). In our detailed point-by-point rebuttal below, we address all comments and highlight the corresponding changes in our revised manuscript.

With that, we believe to have addressed all comments satisfactorily and look forward to hearing whether our manuscript is now acceptable for publication in eNeuro. on behalf of all co-authors, kind regards, Joram D. Mul, PhD.

Assistant Professor Swammerdam Institute for Life Sciences University of Amsterdam - Synthesis of Reviews:

This manuscript presents a network and graph theoretical analysis of male and female mice response to voluntary exercise. The authors use dFOSB labeling across multiple brain regions to infer chronic or cumulative activation within each region. Running resulted in a number of regions with altered activity in both female and male mice and, at the global level, induced sex specific changes in network properties. The manuscript was evaluated by two reviewers who comments are summarized below.

1. The manuscript focuses on stress reactive brain regions and makes statements along the lines of identifying pathways to target to increase stress resilience. However, in the end the manuscript doesn't examine the impact of stress on these networks or show that modulation of any of the impacted brain regions would have an impact on stress related behaviors. The links to stress and brain resilience should be toned down and the main claims should focus on the impacts of exercise, which is what is actually demonstrated here.

Authors response:

We thank the reviewers for this comment and agree we may have overstated our direct links to stress and resilience. We have toned down these and now emphasize the exercise-induced changes in regional ΔFOSB expression and brain-wide network organization (see e.g. our edits on page 3, Introduction).

2. The study used immunolabeling by FOSB, yet the antibody controls supporting the specificity of the serum do not seem to be provided. Further details are needed, even perhaps additional controls if possible (e.g. pre-absorption tests, Western blot, or FOSB IHC in combination with in situ hybridization for Fosb mRNA, eventually tests on knockout tissue).

Authors response:

We agree with the reviewers that antibody specificity is important for correct interpretation of our data. In the present study, we used the well-established and widely used rabbit monoclonal FOSB antibody (5G4; #2251, Cell Signaling Technology), which recognizes both FOSB and ΔFOSB (FOSB2) and Δ2ΔFOSB, but does not cross-react with other FOS proteins, including cFOS, FRA1, and FRA2 (https://www.cellsignal.com/products/primary-antibodies/fosb-5g4-rabbit-monoclonal-antibody/2251). This antibody has been extensively validated before and is widely used (108 citations to date listed by the manufacturer). Specificity of this antibody has been further demonstrated previously using various KO models (e.g. see (Ohnishi et al., 2011).

Due to its prolonged stability, it is in particular ΔFOSB that accumulates after repeated or chronic stimuli. In contrast, FOSB is highly unstable and expressed only transiently. Due to these substantial differences in stability, our experimental design therefore deliberately included an unstimulated period before perfusion to allow degradation of any of the short lived FOSB. Hence, all remaining nuclear immunoreactivity will correspond to accumulated ΔFOSB levels only. This strategy has been applied in previous studies by our own and other labs (e.g. see Hardonk et al., 2026; Mul et al., 2018; Perrotti et al., 2004; Shiba et al., 2025). In the current study, we followed the same approach and locked the running wheels for 24h and left the mice unstimulated before perfusion. We are therefore confident that our signal reflects ΔFOSB. In theory, we cannot fully exclude that possibly, the also short-lived FOSB or Δ2ΔFOSB isoforms may have contributed to our signal, which we have now explicitly stated as a limitation (see page 22).

3. It is claimed that the antigen retrieval was required in the female tissues. This questions the comparability of data: on the one hand, this methodological difference may have masked important sex differences in FOSB activity because the weak female FOSB signal was enhanced by epitope retrieval. It is also possible, that the epitope retrieval increased the magnitude of differences unproportionally in females, and they appear now as real differences, that could exist in males also, but we miss them because they were not magnified by the epitope retrieval. It is unclear why the tissues were differently treated. In particular, why was the epitope retrieval not performed on samples of male mice also? This would have made the male-female comparisons reliable.

Authors response:

The reviewers are correct; this is a relevant and important point that requires clarification.

The female cohort was processed and stained for ΔFOSB after the male cohort. In our initial staining procedure, female brain sections were processed using the same protocol as males (i.e. without antigen retrieval) but showed no detectable ΔFOSB immunolabeling. Antigen retrieval was therefore applied to the female tissues as a necessary step to reveal ΔFOSB epitopes and obtain quantifiable staining to an extent that was comparable to that observed in males. All other aspects of perfusion, fixation, sectioning, antibody concentrations, imaging, and quantification were identical between the two cohorts of different sexes. We did not perform antigen retrieval on the male series because those sections had already been processed and quantified using the standard IHC protocol.

We fully acknowledge that this has introduced a methodological difference between the sexes. This has now been (i) further highlighted in the Methods section (see page. 9), and (ii) we added a statement in the Discussion, explicitly recognizing this as a limitation precluding a direct between-sex comparisons of absolute ΔFOSB levels (see page 22). Importantly, our main conclusions regarding any sex-comparisons are now only based on the within-animal patterns of regional ΔFOSB co-activation and the graph-theoretical metrics derived from the covariance structure across brain regions, rather than on the absolute signal intensity. Antigen retrieval in females is expected to act as a global sensitivity enhancement across regions overall, and therefore should not selectively create or abolish the correlation structure that underlies our network-level analyses. We agree that the difference in staining protocol warrants caution when interpreting absolute male-female differences in ΔFOSB density, but we consider it unlikely that this factor alone accounts for the sex-specific differences present in the network topology that we report. For that reason, we did not directly compare regional differences in ΔFOSB levels between males and females. We have now made this distinction more explicit in the revised manuscript, e.g. see page 22.

4. Considering that several brain areas show a very strong sex hormone sensitivity that is in females strongly estrus cycle phase-dependent, it would be informative to know in what cycle phase were the brains collected, if this is known. Otherwise, it should be stated as an limitation of the study, and be discussed.

Authors response:

We agree with the reviewers that estrous cycle-dependent hormonal fluctuations are an important potential modulator of ΔFOSB expression, which we have reported ourselves as well (also see Shiba et al., 2025). In the present study, we unfortunately did not determine estrous cycle stage at the time of tissue collection and we have now added a statement in the Discussion explicitly acknowledging this as a limitation and note that estrous phase-related variability could have contributed to the observed ΔFOSB patterns in females, predominantly in the sedentary controls (see page 22).

5. The term brain "map" is used a couple of times. While examining 46 areas is indeed an impressive work, it is still not a brain map yet. For instance, the lower brainstem and cerebellum contain also several mood regulation and motor control-related areas that might have been of interest. Claiming that these data form a brain map is an over interpretation (e.g. see page 23 last paragraph and also elsewhere) and should be toned down.

Authors response:

We agree with the reviewers that the terms "brain map" (and "brain-wide") overstated the anatomical coverage of the present study and we have therefore revised the manuscript throughout using more conservative terminology, e.g. see page 2, Significance statement.

Minor point:

The term "expression" is used several times. It is better to say immunoreactivity, immunosignal, abundance of FOSB protein, neuronal activity and other synonyms because genes (or mRNAs) are expressed but not the proteins, and this is a protein level study.

Authors response:

We thank the reviewers and agree. We have revised the manuscript throughout using more appropriate terminology, e.g. see page 1, Abstract.

With that, we believe we have addressed all comments satisfactorily and we look forward to hearing whether our revised manuscript is now acceptable for publication in eNeuro.

Kind regards, on behalf of all co-authors, Joram D. Mul, PhD.

- REFERENCES Hardonk, M. H., Vuuregge, A. H., Hellings, T. P., Eggels, L., Ritsema, W. I. G. R., Lamuadni, K., Unmehopa, U. A., Meerhoff, G. F., Kalsbeek, A., Lucassen, P. J., Schrantee, A., La Fleur, S. E., &Mul, J. D. (2026). Brain-wide induction of ΔFOSB and altered co-activation networks in a rat model for exercise training. Translational Psychiatry, 16(1), 209. https://doi.org/10.1038/s41398-026-03953-3 Mul, J. D., Soto, M., Cahill, M. E., Ryan, R. E., Takahashi, H., So, K., Zheng, J., Croote, D. E., Hirshman, M. F., La Fleur, S. E., Nestler, E. J., &Goodyear, L. J. (2018). Voluntary wheel running promotes resilience to chronic social defeat stress in mice: A role for nucleus accumbens ΔFosB. Neuropsychopharmacology, 43(9), 1934-1942. https://doi.org/10.1038/s41386-018-0103-z Ohnishi, Y. N., Ohnishi, Y. H., Hokama, M., Nomaru, H., Yamazaki, K., Tominaga, Y., Sakumi, K., Nestler, E. J., &Nakabeppu, Y. (2011). FosB Is Essential for the Enhancement of Stress Tolerance and Antagonizes Locomotor Sensitization by ΔFosB. Biological Psychiatry, 70(5), 487-495. https://doi.org/10.1016/j.biopsych.2011.04.021 Perrotti, L. I., Hadeishi, Y., Ulery, P. G., Barrot, M., Monteggia, L., Duman, R. S., &Nestler, E. J. (2004). Induction of ΔFosB in Reward-Related Brain Structures after Chronic Stress. The Journal of Neuroscience, 24(47), 10594-10602. https://doi.org/10.1523/JNEUROSCI.2542-04.2004 Shiba, A., Hardonk, M. H., Foppen, E., Kool, T., La Fleur, S. E., Lucassen, P. J., Yi, C.-X., Stenvers, D. J., Mul, J. D., &Kalsbeek, A. (2025). Voluntary Running and Estrous Cycle Modulate ΔFOSB in the Suprachiasmatic Nucleus of the Wistar Rat. Journal of Circadian Rhythms, 23, 7. https://doi.org/10.5334/jcr.257
